# Life as an evacuee after the Fukushima Daiichi nuclear power plant accident is a cause of polycythemia: the Fukushima Health Management Survey

**DOI:** 10.1186/1471-2458-14-1318

**Published:** 2014-12-23

**Authors:** Akira Sakai, Tetsuya Ohira, Mitsuaki Hosoya, Akira Ohtsuru, Hiroaki Satoh, Yukihiko Kawasaki, Hitoshi Suzuki, Atsushi Takahashi, Gen Kobashi, Kotaro Ozasa, Seiji Yasumura, Shunichi Yamashita, Kenji Kamiya, Masafumi Abe, for the Fukushima Health Management Survey Group

**Affiliations:** Department of Radiation Life Sciences, Fukushima Medical University School of Medicine, 1 Hikarigaoka, Fukushima, 960-1295 Japan; Department of Epidemiology, Fukushima Medical University School of Medicine, Fukushima, Japan; Department of Pediatrics, Fukushima Medical University School of Medicine, Fukushima, Japan; Department of Radiation Health Management, Fukushima Medical University School of Medicine, Fukushima, Japan; Department of Nephrology, Hypertension, Diabetology, and Endocrinology, Fukushima Medical University School of Medicine, Fukushima, Japan; Department of Cardiology and Hematology, Fukushima Medical University School of Medicine, Fukushima, Japan; Department of Gastroenterology and Rheumatology, Fukushima Medical University School of Medicine, Fukushima, Japan; Department of Planning and Management, National Institute of Radiological Sciences, Chiba, Japan; Department of Epidemiology, Radiation Effects Research Foundation, Hiroshima, Japan; Department of Public Health, Fukushima Medical University School of Medicine, Fukushima, Japan; Radiation Medical Science Center for the Fukushima Health Management Survey, Fukushima Medical University School of Medicine, Fukushima, Japan; Japan and Atomic Bomb Disease Institute, Nagasaki University, Nagasaki, Japan; Department of Experimental Oncology, Research Institute for Radiation Biology and Medicine, Hiroshima University, Hiroshima, Japan

**Keywords:** Evacuee, Polycythemia, Fukushima Daiichi Nuclear Power Plant accident, Lifestyle-related disease, The Fukushima Health Management Survey

## Abstract

**Background:**

The Great East Japan Earthquake and the Fukushima Daiichi nuclear disaster forced people to evacuate their hometowns. Many evacuees from the government-designated evacuation zone were forced to change their lifestyle, diet, exercise, and other personal habits. The Comprehensive Health Check (CHC), 1 of 4 detailed surveys of The Fukushima Health Management Survey (FHMS), was implemented to support the prevention of lifestyle-related disease. The aim of this study was to analyze changes in red blood cell count (RBC), hemoglobin (Hb) levels, and hematocrit (Ht) levels by comparing data from the medical health checkup before and after the disaster in individuals who were 40 years old or older.

**Methods:**

Subjects in this study were Japanese men and women living in the vicinity of the Fukushima Daiichi Nuclear Power Plant in Fukushima prefecture. Annual health checkups with a focus on metabolic syndrome for insured persons/dependents aged 40 or older by Health Care Insurers have been conducted since 2008. All analyses in this study were limited to men and women aged 40–90 years. Changes in RBC, Hb levels, Ht levels, and prevalence of polycythemia before and after the disaster were compared.

**Results:**

First, RBC, Hb, and Ht significantly increased in both men and women evacuees. The evacuation was significantly associated with increased Hb levels after adjustment for age, gender, smoking status, excess ethanol intake, BMI, and baseline Hb level (β = 0.16, *p* < 0.001). Furthermore, the prevalence of polycythemia stratified by smoking status or obesity also increased in the evacuee group.

**Conclusions:**

To our knowledge, this is the first report revealing that the evacuation was associated with the risk of polycythemia. This information could be very important for periodic health checkup and lifestyle recommendations for evacuees in the future.

**Electronic supplementary material:**

The online version of this article (doi:10.1186/1471-2458-14-1318) contains supplementary material, which is available to authorized users.

## Background

The Great East Japan Earthquake that occurred on 11 March 2011 and the Fukushima Daiichi nuclear disaster forced people to evacuate their hometowns with notice, caused them to change their lifestyle to fit a completely new situation, and produced anxiety about radiation. In response to concerns about the effects these factors could have on health, the Comprehensive Health Check (CHC), 1 of 4 detailed surveys of the Fukushima Health Management Survey (FHMS), was implemented to support the early detection and treatment of disease as well as the prevention of lifestyle-related diseases, such as heart disease and cerebrovascular disease.

Japan experienced atomic bombings in Hiroshima and Nagasaki in 1945. In 1947, the Atomic Bomb Casualty Commission (ABCC) was established to investigate the health impacts on atomic bomb (A-bomb) survivors. Later, a large-scale cohort study of survivors was started in order to investigate the long-term stochastic effects of radiation. The ABCC had continued follow-up surveys through the present time [1–3]. In April 1986, the worst nuclear disaster in human history occurred at the Chernobyl Nuclear Power Plant. The accident released a large quantity of radioactive material into the atmosphere. The USSR Ministry of Health started the Russian National Medical and Dosimetric Registry in June the same year to resister residents exposed to radiation. However, an epidemiologic study evaluating long-term radiation effects on public health was unfortunately not implemented soon enough after the accident [4].

The primary purposes of the FHMS are to monitor the long-term health of residents, promote their future well-being, and determine whether long-term low-dose radiation exposure has health effects. The FHMS consists of a basic survey and 4 detailed surveys, namely, thyroid ultrasound examination, comprehensive health check, a mental health and lifestyle survey, and a pregnancy and birth survey [5]. Many evacuees from the government-designated evacuation zone were forced to change their lifestyle, diet, exercise, and other personal habits. Some could not receive adequate health checks, and some had anxieties about their health [6]. The CHC attempts to review their health information, assess the incidence of various diseases, and improve their health status. Here we focused on changes in red blood cell count before and after the disaster.

## Methods

### Subjects

Subjects in this study were Japanese men and women living in the vicinity of the Fukushima Daiichi Nuclear Power Plant in Fukushima prefecture; Tamura, Minami-Soma, Kawamata, Hirono, Naraha, Tomioka, Kawauchi, Okuma, Futaba, Namie, Katsurao, Iitate, and Date. All residents of Hirono, Naraha, Tomioka, Kawauchi, Okuma, Futaba, Namie, Katsurao, and Iitate, and part of residents of Tamura, Minami-Soma, Kawamata, and Date were forced to evacuate their homes due to the governmental direction after the disaster. In these communities, annual health checkups with a focus on metabolic syndrome for insured persons/dependents aged 40 or older by Health Care Insurers have been conducted since 2008. All analyses in this study were limited to men and women aged 40–90 years. Between 2008 and 2010, 41,633 men and women (18,745 men and 22,888 women, mean 67 years) in the communities participated in the health checkups. The initial exclusion criteria were persons without peripheral blood hemoglobin (Hb) data (n = 23,279) and those with a past history of or who were being treated for hematologic disease or residents undergoing dialysis due to renal impairment (n = 107). The remaining data of 18,247 men and women (7,647 men and 10,709 women, mean 68 years) were used for the analyses as a baseline data. Informed consent was obtained from the community representatives to conduct an epidemiological study based on guidelines of the Council for International Organizations of Medical Science [7]. This study was approved by the Ethics Committee of the Fukushima Medical University School of Medicine (approval number 1916).

Follow-up examinations were conducted between 2011 and 2012 as a part of the CHC. Detailed methods of the CHC was described by Yasumura previously [5]. Basically, this CHC performed health examinations for individuals of all ages living in the evacuation zone designated by the government, who were officially registered residents at the time of the earthquake. 10,718 men and women (4,627 men and 6,091 women, follow-up rate: 59%) received the follow-up examination after the disaster, and an average follow up was 1.6-year. There were some differences in baseline characteristics between individuals who received follow-up examinations and those who did not, such as mean age (67.4 vs 69.7 years) and prevalence of diabetes mellitus (10.3% vs 12.4%) and hypertension (55.5% vs 60.2%), while there were no differences in baseline BMI and smoking status. Baseline characteristics of individuals who received the follow-up examinations are shown in Table 1.

**Table 1:** **Means or prevalence for baseline characteristics of participants in this study**

	Total (n = 10,718)				
Baseline characteristics	Evacuees		Non—evacuees		*p*value***
n	7,446 3		3,272		
Age (years)	66.3	±9.8**	69.8	±8.8**	0.01
Sex (% women)	56.0		58.8		<0.01
Body mass index (kg/m^2^)	23.7	±3.3**	23.4	±3.3**	<0.01
Overweight* (%)	31.8		28.9		<0.01
Hypertension (%)	54.5		57.7		<0.01
Diabetes Mellitus (%)	10.7		9.4		0.04
Current smoker (%)	12.8		10.2		<0.001
Current drinker (%)	23.7		20.5		<0.001

*Body mass index ≥ 25.0 kg/m^2^; **Standard deviaion; ***Student t—tests or chi—squared tests.

### Measurements

Individuals aged 16 years or older are evaluated according to items in the Specific Health Examination based on the Act on Assurance of Medical Care for Elderly People (Act No. 80, 1982). The items are listed in Additional file 1: Table S1. Additional items for assessment include serum creatinine (Cr), estimated glomerular filtration rate (eGFR), uric acid (UA), urine testing for occult blood, and peripheral blood count, which includes red blood cell count (RBC), hematocrit (Ht), Hb, platelet count, and white blood cell (WBC) count with subpopulations of white cells. All peripheral blood cell counts before and after the disaster were measured using the Autoanalyzer XN9000 (Sysmex Co. Inc., Kobe, Japan) at the laboratory of the Fukushima Preservative Service Association of Health, except for residents in Futaba (n = 702). Since the trends in blood cell counts before and after the disaster in Futaba were essentially same as other communities, we included the data of Futaba to analyze. In the CHC, the definition of anemia is Hb less than 13.0 g/dL in men and less than 12.0 g/dL in women. The definition of polycythemia is Hb of 18.0 g/dL or more in men and 16.0 g/dL or more in women.

### Statistical analysis

Means or prevalence for baseline variables of interest were compared between the evacuees (n = 7,446) and non-evacuees (n = 3,272) using Student t-tests or chi-squared tests. Changes in RBC, Hb levels, Ht levels, and prevalence of polycythemia before and after the disaster were compared using a Student’s paired *t* test or McNemer’s test. Analysis of covariance (ANCOVA) was used to examine the differences in the variables between the evacuees and non-evacuees adjusted for age (years), gender, and smoking status. Multiple linear regression analysis was used to test for associations of evacuation and other potential confounders with change in Hb, and the fit of models was tested using residual analysis. The potential confounders were age (years), gender, smoking status, excess ethanol intake (≥44 g/day), BMI, and baseline Hb level (Model 1), and further adjustment for change in BMI before and after the disaster (Model 2). Furthermore, logistic regression analysis was used to test for association of evacuation with newly-developing polycythemia after the disaster adjusting for the potential confounders.

SAS version 9.3 (SAS Institute, Cary, North Carolina, USA) was used for analyses. All probability values for statistical tests were 2-tailed and *p* values of less than 0.05 were regarded as statistically significant.

## Results and discussion

On implementing the CHC, we expected that there were no individuals with the risk of cytopenia according to the results of estimation of external dose in the FHMS [8, 9]. Furthermore, because the comparable items in the peripheral blood count before and after the disaster were RBC, Hb, and Ht, we analyzed the change in those items. Changes in lifestyle among evacuees caused increases in body weight and blood pressure [10]. Therefore, we expected that it would cause polycythemia as well. The standard values for peripheral blood in the CHC are as follows: RBC 400–579 × 10^4^/μL, Hb 13.1-17.9 g/dL, and Ht 38.0-54.9% in men; RBC 370–549 × 10^4^/μL, Hb 12.1-15.9 g/dL, and Ht 33.0-47.9% in women. As for the diagnosis of polycythemia, one of these items is beyond the standard value.

First, RBC, Hb, and Ht significantly increased in both men and women evacuees (Table 2). Furthermore, age-adjusted *p* value for comparing the changes of these items in the evacuee group to those in the non-evacuee group between before and after the earthquake was significant (Table 2). Next we performed a multivariate analysis to find whether the evacuation was an independent factor for an increase in Hb in the presence of smoking, one of the important causes of polycythemia, and obesity and weight gain, which were already reported to be related to the evacuation [9]. The evacuation was significantly associated with increased Hb levels after adjustment for age, gender, smoking status, excess ethanol intake, BMI, and baseline Hb level (β = 0.16, *p* < 0.001). In the present study, mean levels of BMI of the participants increased from 23.6 kg/m^2^ to 24.0 kg/m^2^ before and after the disaster (*p* < 0.001). The association between evacuation and increased Hb levels was attenuated after further adjustment for change in BMI before and after the disaster, but it remained statistically significance (β = 0.11, *p* < 0.001). Furthermore, increasing of Hb levels in the present study might not be influenced by change in proportion of smoking status because the proportion of smokers decreased after the disaster in both evacuees and non-evacuees; from 12.8% to 11.9% for evacuees and from 10.2% to 8.4% for non-evacuees.

**Table 2:** **Changes in RBC, Hb, and Ht according to evacuation status**

Sex	Evacuation	N	RBC (average) (x 10 ^4^/*μ*L)			∆	*p*	Hb (average) (g/dL)			∆	*p*	Ht (average) (%)			∆	*p*
			Before		After			Before		After			Before		After		
Men	(-)	1349	463.1		461.3	-1.8	0.02	14.46		14.57	0.10	<0.0001	43.57		43.45	-0.12	0.11
	(+)	3278	472.7		479.2	6.5	<0.0001	14.77		15.05	0.28	<0.0001	44.34		44.74	0.40	<0.0001
				p*		<0.0001			p*		<0.0001			p*		<0.0001	
Women	(-)	1923	434.5		432.2	-2.3	<0.0001	13.04		13.15	0.11	<0.0001	40.00		39.95	-0.05	0.31
	(+)	4168	439.6		443.1	3.5	<0.0001	13.18		13.40	0.22	<0.0001	40.42		40.74	0.33	<0.0001
				p*		<0.0001			p*		<0.0001			p*		<0.0001	

*Age—adjusted p value for comparing changes in the evacuee group to changes in the non-evacuee group before and after the earthquake.

RBC, red blood cell count: Hb, hemoglobin; Ht. hematocrit.

In this study the standard values of obesity and the significant weight gain are a BMI of 25 kg/m^2^ or more and an increase in BMI of 1 kg/m^2^ or more, respectively. We also analyzed age-adjusted *p* values for comparing the changes in Hb in the evacuee group to those in the non-evacuee group between before and after the earthquake because the value of Hb is a representative index of polycythemia (Table 3). In men, the evacuation significantly influenced an increase in Hb regardless of smoking status, and the group that did not smoke showed a more significant increase. In the relationship between the changes in Hb and BMI or increase in BMI, the evacuation had a significant influence on the group with BMI less than 25 kg/m^2^ and increase of less than 1 kg/m^2^ in BMI. In women, the evacuation significantly influenced an increase in Hb in the group that did not smoke. The evacuation increased the value of Hb regardless of obesity, and the increase in Hb was significantly higher in the group with BMI less than 25 kg/m^2^. Furthermore, the evacuation was followed by a significant increase in Hb in the group with less than 1 kg/m^2^ increase in BMI. These results revealed that life as an evacuee leads to increase in Hb. However, what is especially important is whether an incidence of polycythemia in the evacuee group, but not in the non-evacuee group, significantly increased after the disaster. Prevalence of polycythemia before and after the disaster were 0.79% and 1.16% for the non-evacuees (*p* = 0.10) and 0.89% and 1.54% for the evacuees (*p* < 0.001), respectively. Then, we further analyzed the prevalence of polycythemia stratified by smoking status or obesity, the tendency was virtually unchanged (Table 4). Among smokers, prevalence of polycythemia before and after the disaster were 1.19% and 1.79% for the non-evacuees (*p* = 0.50) and 1.89% and 3.67% for the evacuees (*p* < 0.01), and among non-smokers, those of polycythemia were 0.75% and 1.09% for the non-evacuees (*p* = 0.16) and 0.74% and 1.23% for the evacuees (*p* < 0.001), respectively. Among obese, prevalence of polycythemia before and after the disaster were 1.06% and 1.59% for the non-evacuees (*p* = 0.36) and 1.48% and 2.45% for the evacuees (*p* < 0.01), and among non-obese, those of polycythemia were 0.69% and 0.99% for the non-evacuees (*p* = 0.23) and 0.61% and 1.12% for the evacuees (*p* < 0.001), respectively. Based on these results, we suggest that the evacuation is an independent factor for polycythemia regardless of smoking status, obesity, or weight gain, while an logistic regression analysis showed the association between evacuation and newly-developing polycythemia did not reach statistical significance; the multivariable adjusted odds ratio for evacuation is 1.11 (95% confidence interval; 0.71-1.73).

**Table 3:** **Influence of the evacuation on changes of Hb in relation to smoking, obesity, and weight gain**

Sex	Factors	n	Changes in Hb (g/d)		p*
			Evacuation (-)	Evacuation (+)	
Men	Smoking (-)	3,579	0.11	0.29	<0.0001
	Smoking (+)	1,048	0.09	0.27	0.008
	BMI <25 kg/m^2^	3,180	0.10	0.30	<0.0001
	BMI 25 ≧ kg/m^2^	1,447	0.13	0.24	0.08
	Change in BM < 1 kg/m^2^	2,981	0.03	0.15	0.006
	Change in BM ≧ 1 kg/m^2^	1,646	0.44	0.46	0.89
Women	Smoking (-)	5,851	0.12	0.22	<0.0001
	Smoking (+)	240	-0.04	0.13	0.12
	BMI <25 kg/m^2^	4,229	0.12	0.22	0.0002
	BMI 25 ≧ kg/m^2^	1,862	0.10	0.21	0.02
	Change in BM < 1 kg/m^2^	4,444	0.09	0.17	0.002
	Change in BM ≧ 1 kg/m^2^	1,647	0.22	0.31	0.13

*Age—a djusted *p* value for comparing changes in the evacuee group to changes in the non—evacuee group before and after the earthquake.

BMI, body math index; Hb, hemoglobin.

**Table 4:** **Influence of the evacuation on prevalence of polycythemia in relation to smoking and obesity**

				Before	After	p*
			n	Number of polycythemia (%)	Number of polycythemia (%)	
Total		Evaculation (-)	3,272	26 (0.79)	38 (1.16)	0.10
		Evaculation (+)	7,446	66 (0.89)	115 (1.54)	<0.001
Obese	+	Evaculation (-)	945	10 (1 .06)	1 5 (1.59)	0.36
		Evaculation (+)	2,364	35 (1 .48)	58 (2.45)	<0.01
	-	Evaculation (-)	2,327	6 (0.69)	23 (0.99)	0.23
		Evaculation (+)	5,082	31(0.61)	57 (1.12)	<0.001
Smoking	+	Evaculation (-)	335	4 (1.19)	6 (1.79)	0.50
		Evaculation (+)	953	18 (1 .89)	35 (3.67)	0 < 0.01
	-	Evaculation (-)	2,973	22 (0.75)	32 (1.09)	0.16
		Evaculation (+)	6,493	48 (0.74)	80 (1.23)	<0.001

*McNemar’s test for comparing changes in the prevalence of polycythemia in the evacuee and non—evacuee group before and after the earthquake.

Common causes of polycythemia are polycythemia vera (myeloproliferative disease), secondary polycythemia caused by diseases such as pulmonary heart disease that induce a chronic lack of oxygen or an erythropoietin-producing tumor, and relative polycythemia or stress-induced polycythemia. The mechanism of stress-induced polycythemia is unknown, and it is usually diagnosed in middle-aged men who smoke, are obesity, and have hypertension, or hyperuricemia. Although dehydration induced by use of diuretic for hypertension might affect Hb level, the difference of such a factor between evacuees and non-evacuees could not be clarified. To our knowledge, this is the first report showing that evacuation is a cause of polycythemia. Even in the group that was not evacuated, the value of Hb before and after the disaster increased significantly in both men and women (Table 2). Some kind of stress induced by the disaster might be causing the increase in Hb in this group.

The increase of lifestyle-related disease was expected because obesity, weight gain, and high blood pressure were shown to occur in evacuees after the disaster, and this investigation revealed that life as an evacuee causes polycythemia. Here we suggest that periodic health checkups and lifestyle guidance for evacuees in the future is very important.

## Conclusions

In summary, RBC, Hb, and Ht significantly increased in both men and women evacuees. Furthermore, the prevalence of polycythemia also increased in the evacuee group. Periodic health checkups and lifestyle guidance should be carefully planned for evacuees.

## Appendix

### The Fukushima Health Management Survey Group

Masafumi Abe, Shunichi Yamashita, Kenji Kamiya, Seiji Yasumura, Mitsuaki Hosoya Akira Ohtsuru, Akira Sakai, Shinichi Suzuki, Hirooki Yabe, Masaharu Maeda, Shirou Matsui, Keiya Fujimori, Tetsuo Ishikawa, Tetsuya Ohira, Tsuyoshi Watanabe, Shigeatsu Hashimoto, Kenneth Eric Nollet, Shinichi Niwa, Yoshisada Shibata.

## Electronic supplementary material

Additional file 1: Table S1: Items included in comprehensive health check. (XLSX 45 KB)

## References

[1] *The Radiation Effects Research Foundation*. A brief description available from: http://www.rerf.or.jp/shared/briefdescript/briefdescript.pdf

[2] Shigematsu I (1992). Ionizing radiation and health. J Epidemiol.

[3] Kodama K, Mabuchi K, Shigematsu I (1996). A long-term cohort study of the atomic-bomb survivors. J Epidemiol.

[4] Bennett B, Repacholi M, Carr Z (2006). Health effects of the Chernobyl accident and special health care programmes. Report of the UN Chernobyl Forum Expert Group “Health”.

[5] Yasumura S, Hosoya M, Yamashita S, Kamiya K, Abe M, Akashi M, Kodama K, Ozasa K for the Fukushima Health Management Survey Group (2012). Study protocol for the Fukushima Health Management Survey. J Epidemiol.

[6] Sugimoto A, Krull S, Nomura S, Morita T, Tsubokura M (2012). The voice of the most vulnerable: lessons from the nuclear crisis in Fukushima, Japan. Bull World Health Organ.

[7] **International guidelines for ethical review of epidemiological studies** *Law Med Health Care* 1991,**19**(3–4)**:**247–258. 1779694

[8] Nagataki S, Takamura N, Kamiya K, Akashi M (2013). Measurements of individual radiation doses in residents living around the Fukushima nuclear power plan. Radiation Res.

[9] Tenth YS, Warrent K (2014). Sinclair Keynote address: the Fukushima nuclear power plant accident and comprehensive health risk management. Health Phys.

[10] *Fukushima Radiation and Health*. Survey results available from http://www.fmu.ac.jp/radiationhealth/results/

[CR11] The pre-publication history for this paper can be accessed here: http://www.biomedcentral.com/1471-2458/14/1318/prepub

